# Asymmetric transformations from sulfoxonium ylides

**DOI:** 10.1039/d1sc05708a

**Published:** 2021-12-08

**Authors:** Clarice A. D. Caiuby, Lucas G. Furniel, Antonio C. B. Burtoloso

**Affiliations:** São Carlos Institute of Chemistry, University of São Paulo São Carlos SP CEP 13560-970 Brazil antonio@iqsc.usp.br

## Abstract

Sulfoxonium ylides are important surrogates for diazo compounds, and their use in industry as safer alternatives has been evaluated during recent years. Beyond the known classical transformations, these ylides have also been used in a surprising plethora of novel and intrinsic chemical reactions, especially in recent years. Bench stability and handling are also an advantage of this class of organosulfur molecules. Despite this, efficient asymmetric transformations, specifically catalytic enantioselective versions, have only recently been reported, and there are specific reasons for this. This perspective article covers this topic from the first studies up to the latest advances, giving personal perspectives and showing the main challenges in this area in the coming years.

## Abstract 2

Asymmetric transformations involving sulfoxonium ylides, culminating in catalytic enantioselective versions, are discussed in this perspective.

## Graphical abstract

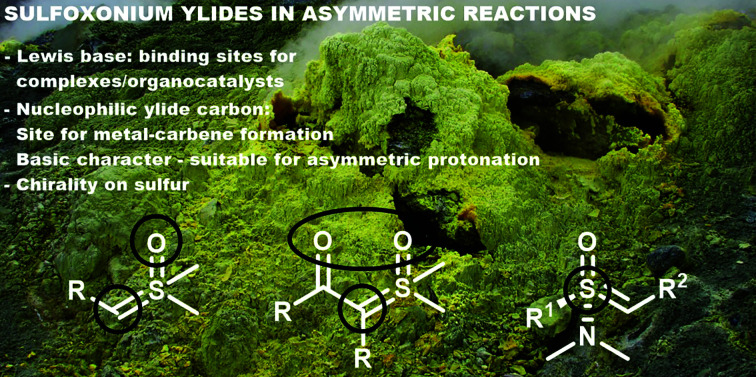

## 1. Introduction

Since the late 1960s, and the pioneer work of Corey and Chaykovsky,^1–3^ sulfur ylides have come a long way in the area of organic synthesis. Their applicability, usually as surrogates for diazo compounds, has now received recognition as highly versatile substrates in numerous chemical transformations. Owing to some of their inherent properties, specifically associated to the α-electron withdrawing group (EWG) stabilized sulfur ylides, such as the thermal stability, low toxicity, ease of use and long shelf life, this class of compounds has been effectively applied in large scale reactions and has attracted attention from industry.^4–11^

**Scheme 1:**
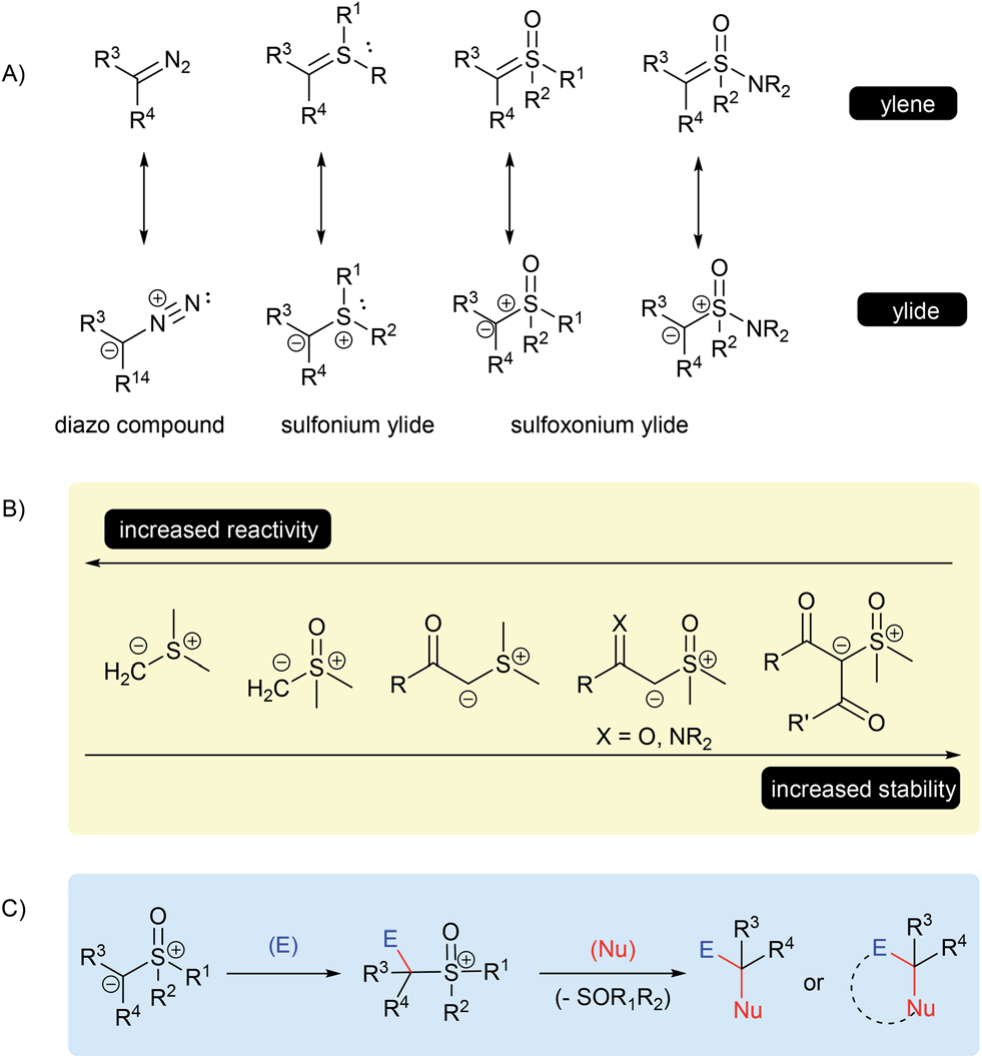
Structure and stability of sulfur ylides.

In general, ylides can be described as having two main canonical forms, ylide and ylene, however, the dipolar structure, with a carbanion bounded to a positive sulfur moiety, plays a major role in describing the real structure of the sulfur ylides (Scheme 1A).^12–15^ These structural features are important to understanding their unique reactivity based on three main points: the C-nucleophilicity of the carbanion center; the leaving-group character of the sulfur group; and the overall structural stability, which depends on the ability of adjacent substituent groups to stabilize the negative charge of the carbanion, as well as on the substitution of the sulfur atom (Scheme 1B). For example, a solution in THF under an inert atmosphere of dimethylsulfonium methylide readily decomposes at room temperature, while dimethylsulfoxonium methylide (DMSM) under the same conditions can be stored and remain stable for several days.^2^ On the other hand, α-carbonyl sulfoxonium ylides are usually crystalline bench-stable solids, the delocalization of the negative charge through an EWG significantly contributes to its stability. Amino-sulfur ylides, first described in 1968 by Johnson, containing an –NR_2_ substituent at the sulfur atom, have a similar or enhanced stability compared to the parental *S*-alkyl-substituted sulfur ylide.^16,17^

Therefore, these characteristics make sulfur ylides valuable substrates for bi-functionalization reactions, as this species contains a nucleophilic site neighboring a good leaving group moiety, all attached within the same structure.

**Scheme 2:**
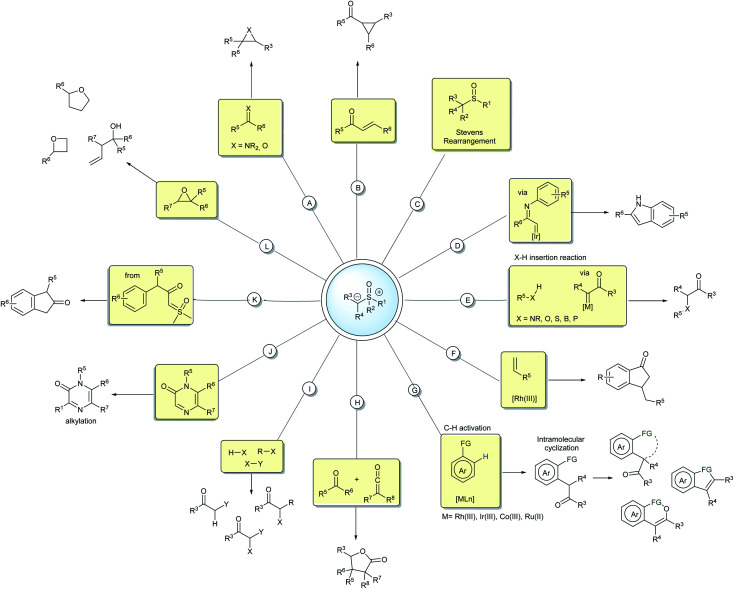
Representative examples of the reactions of sulfoxonium ylides.

The mechanism for these transformations usually occurs *via* the nucleophilic addition of the ylide carbanion to an electrophile with subsequent displacement of the positive sulfur group through a nucleophilic substitution. When an external nucleophile is applied, the reaction can afford α,α-bis-functionalized products, but if an intramolecular nucleophile is available in the intermediate structure, it results in a cyclization process (Scheme 1C). Epoxidation, aziridination, cyclopropanation, Steven's rearrangements and sigmatropic rearrangements are the main classical reactions that have been established for the sulfur ylides (Scheme 2A–C).^18,19^

In the last few decades, sulfoxonium ylides have regained the attention of many researchers and those in the industry, particularly owing to their ability to generate carbene species, a field that was for a long time dominated by diazo compounds. Metal-carbenes from sulfoxonium ylides have been employed in several insertion reactions, such as into X–H (N, O, S, B, P) and C–H bonds (Scheme 2D and E).^20–25^ These ylides can also participate in cross-coupling reactions by metal-carbene migratory insertion, resulting in C–H activation processes mediated mainly by the M(iii) catalysts.^26,27^ From the products provided in the C–H activation step, many post functionalization reactions can be performed to generate structurally diverse heterocycles and carbocycles (Scheme 2F and G).^28–36^ Important progress has also been made with regards to metal or carbene-free reactions by exploring the potential nucleophilicity of the sulfoxonium ylides. Some representative examples are the insertion reactions,^37–39^ α-halogenations,^40^ alkylations,^41^ cyclizations,^42–45^ ring CH_2_-homologations and epoxide ring opening reactions (Scheme 2H–L).^46–48^

With respect to the asymmetric transformations, the reactions of the sulfoxonium ylides still lack research compared to the sulfonium ylides. For example, numerous literature reviews have been published describing the applications of sulfonium ylides and their asymmetric transformations.^49–51^ This fact can be attributed to several factors, for example, sulfonium salts, the main precursors in the syntheses of sulfonium ylides, are readily available from the alkylation of thioethers and can be prepared with a wide structural diversity. In addition, these substrates demonstrate a good behavior in the presence of metal complexes and organocatalysts, and have been successfully applied in asymmetric reactions mediated by covalent and H-bonding catalysis,^52–54^ metal-carbene formation^55^ and Lewis and Brønsted acid catalysts.^56,57^ In the case of sulfoxonium ylides, although these are more stable and attractive, the difficulty in accessing a vast number of structurally different compounds has limited their use over the years. For example, the preparation of the precursors, the sulfoxonium salts, are basically limited to the methylation of dimethyl sulfoxide (the S alkylation of dimethyl sulfoxide cannot be performed efficiently with other electrophiles compared to methyl iodide nor any alkylation of a different sulfoxide); this limits the degree of substitution around the sulfur. The other two main methods that circumvent this, include the oxidation of sulfonium salts and the reaction of sulfoxides with diazo compounds catalyzed by transition metals, these will be discussed in detail below. Unfortunately, neither of these two methods are efficient or attractive for large scale use as yet. Considering this, the majority of structurally different sulfoxonium ylides prepared to date have been derived from the acylation of dimethyl sulfoxonium methylide. In addition to some limitations regarding their synthesis, the presence of the more electronegative oxygen atom bound to the sulfur results in two major differences in the reactivity. First, it creates a Lewis base coordinate site, which can interfere in a catalytic cycle.^58–60^ Second, the more positive sulfur atom leads to better stabilization of the adjacent negative charge by the electrostatic effect, making this compound less reactive and less C-nucleophilic when compared to the corresponding sulfonium ylide. This paragraph illustrates the importance of the development of novel methods to synthesize sulfoxonium ylides and that there is still room for further research in this area.

Based on this perspective, we intend to demonstrate the importance and applicability of sulfoxonium ylides in organic synthesis, culminating in the recent and novel enantioselective transformations. After the above brief introduction to the topic, the article will continue with the importance and appeal of sulfoxonium ylides to the industry. Methods to prepare these ylides, including synthetic routes to obtain pro-chiral sulfoxonium ylides for asymmetric transformations, will be covered before the advances in asymmetric and catalytic enantioselective transformations are described.

## 2. Sulfoxonium ylides in industry: importance and applications

**Scheme 3:**
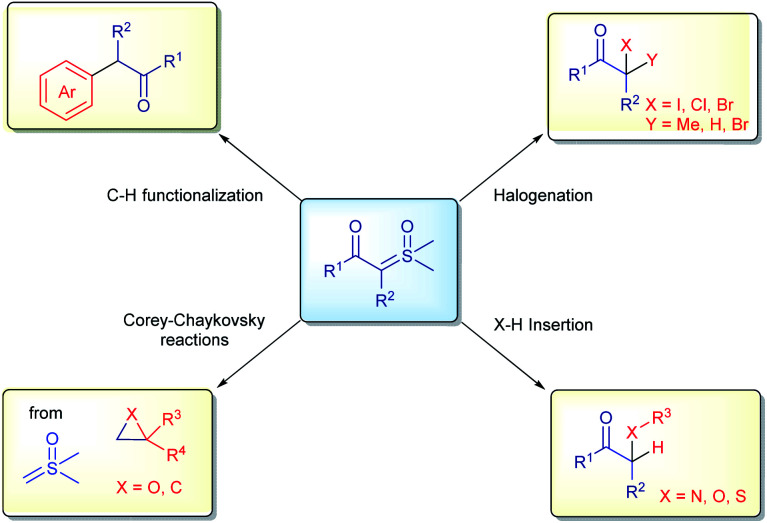
General applications of sulfoxonium ylides in industry.

Diazo compounds, when compared to sulfur ylides, have some drawbacks related to their preparation (specifically when diazomethane needs to be employed), the thermal stability and nature of the leaving group in the reactions (N_2_ gas).^61,62^ In order to overcome these unwanted aspects of diazo chemistry, especially in large scale preparations, sulfur ylides have not only emerged as safer substitutes, but also possess a unique reactivity in organic synthesis. Furthermore, sulfoxonium ylides are even more advantageous, owing to the additional benefit of their enhanced stability, their chemical reactions release sulfoxides as by-products, which are less toxic, non-volatile and odorless compared to the sulfides derived from the reactions with sulfonium ylides, these characteristics are highly desirable in industrial process. Within this topic, the application of sulfoxonium ylides in industry will be presented, highlighting some of the advantages, limitations and, when relevant, making comparisons with the correlated diazo compounds. The content is categorized by the type of reaction: halogenation reactions, X–H insertions, C–H functionalization, Corey–Chaykovsky reactions (Scheme 3) and the synthesis of vinyl sulfoxonium ylides.

**Scheme 4:**
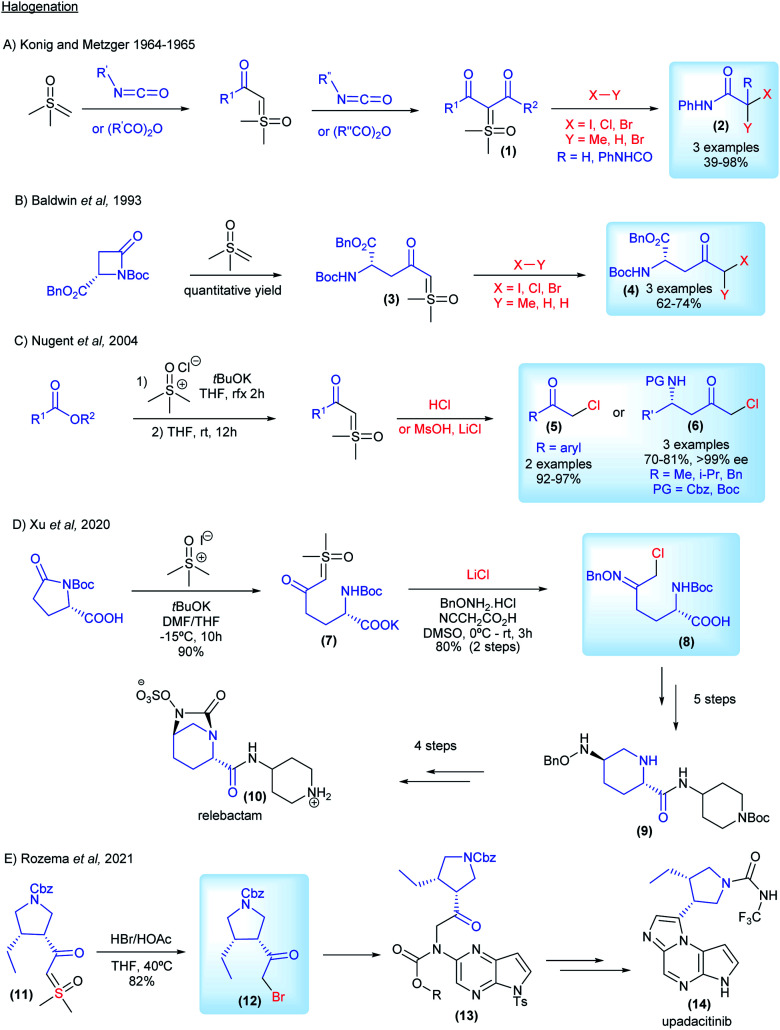
Industrial research and applications relating to sulfoxonium ylides in halogenation reactions.

The interest from industry in preparing halo-carbonyl compounds from sulfoxonium ylides started in 1964, when König and Metzger from BASF reported the synthesis of di- and monocarbonyl sulfoxonium ylides from anhydrides and isocyanates (1, Scheme 4A). They applied these compounds as substrates for difunctionalization reactions using MeI, HCl and Br_2_ to obtain the corresponding halogenated products in 39–98% yields (2, Scheme 4A).^63,64^ Almost 30 years later, Baldwin and co-workers from ICI Agrochemicals, used ketosulfoxonium ylides 3 derived from β-lactams to form functionalized γ-keto-α-amino acids (4, Scheme 4B).^65^ In 2004, Nugent and co-workers from Bristol-Myers Squibb also reported the halogenation of ketosulfoxonium ylides.^66,67^ In their article the authors not only placed the emphasis on the importance of the α-chloroketones for the synthesis of important pharmaceuticals, such as HIV protease inhibitors, but also on the drawbacks of using diazo compounds in their preparation and the ability of sulfoxonium ylides to act as competent surrogates for diazo compounds. The authors were able to transform α-ketosulfoxonium ylides (derived from amino acids) into the corresponding α-haloketones with no loss of enantiopurity (6, Scheme 4C). In 2010, researchers from Codexis Inc. also showed an interest in the preparation of α-chloroketones from sulfoxonium ylides, in particular enantiomerically pure α-chloroketones from ketosulfoxonium ylides derived from amino acids, using a similar procedure.^68^ Lastly, in 2020, researchers from Merck disclosed the diastereoselective synthesis of the oxime ether 9, which is an intermediate in the synthesis of the β-lactamase inhibitor relebactam 10. The authors also utilized the halogenation methodology to synthesize chloroketone (8) from the corresponding sulfoxonium ylide (7), which was obtained from (*S*)-*N*-Boc pyroglutamic acid (Scheme 4D). It is important to note that the reaction was carried out at a 50 g scale, showcasing its robustness.^69^ Very recently, in 2021, Rozema and co-workers from AbbVie, employed the halogenation methodology to synthesize a bromo-ketone intermediate (12, Scheme 4E), from the corresponding sulfoxonium ylide (11). This halogenation procedure was carried out at a large scale, using 300 kg of sulfoxonium ylide as the starting material. This key intermediate was used in the synthesis of upadacitinib (JAK inhibitor) (14).^70^

**Scheme 5:**
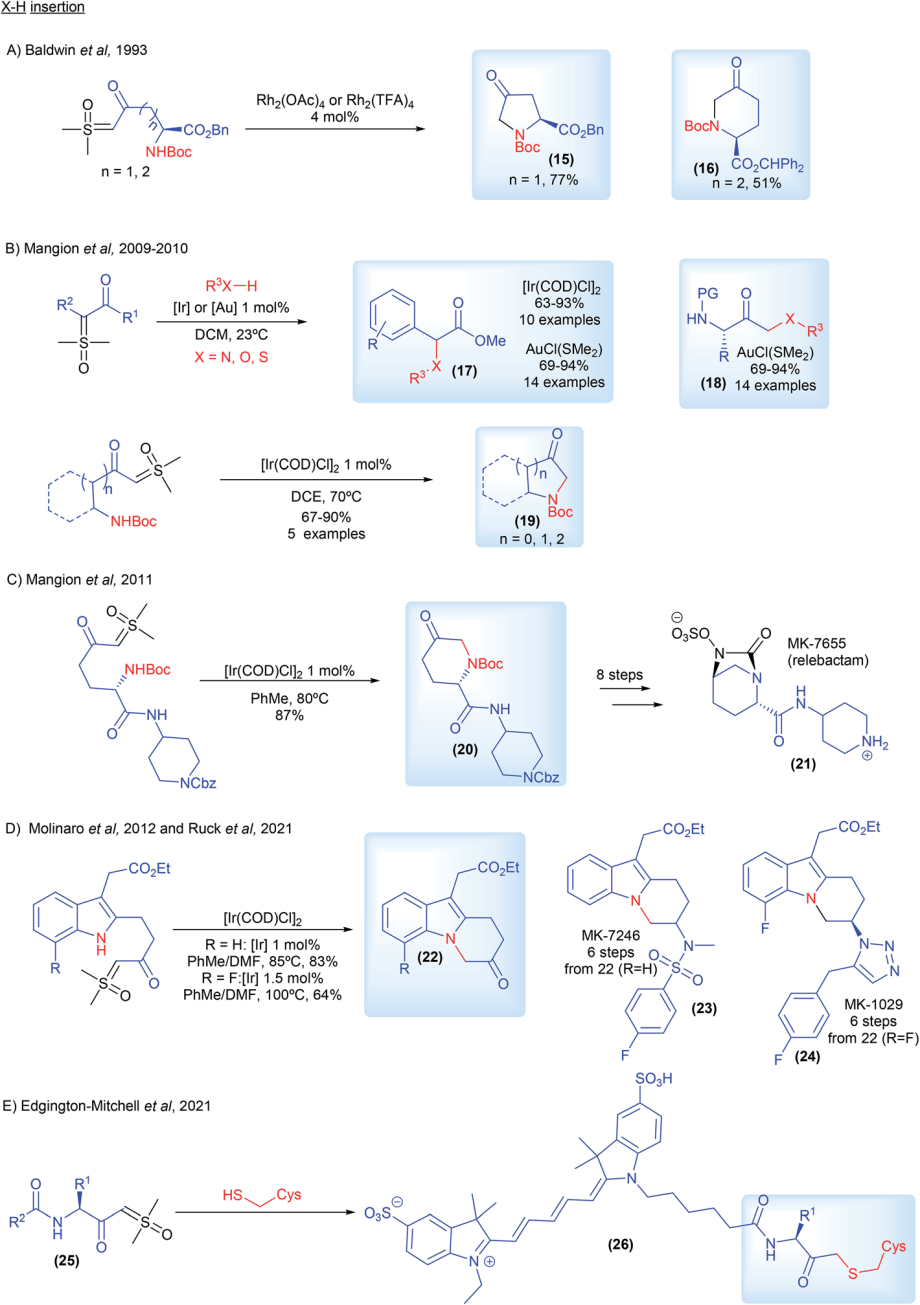
Industrial research and applications relating to sulfoxonium ylides in X–H insertion reactions.

The first example of carbene mediated N–H insertion with sulfoxonium ylides was demonstrated by Baldwin and co-workers from ICI Agrochemicals.^71^ In 1993, they developed a Rh_2_(TFA)_4_ catalyzed intramolecular methodology to provide access to non-proteinogenic amino acid derivatives 15 and 16 (Scheme 5A). Interested in surrogates for diazo compounds, Mangion and co-workers from Merck and Co., published back to back articles in 2009 and 2010 in which they screened several transition metal catalysts in a detailed study, establishing [Ir(COD)Cl]_2_ and AuCl(SMe_2_) as catalysts of choice for X–H insertion with sulfoxonium ylides (first general X–H insertion methodology with sulfoxonium ylides, 17–19, Scheme 5B).^20,72^ In the following year the same group from Merck applied this recently developed methodology in the synthesis of MK-7655 (relebactam) (21, Scheme 5C). Starting from the corresponding ketosulfoxonium ylide, intermediate 20 was obtained in an 87% yield *via* [Ir(COD)Cl]_2_ catalyzed intramolecular N–H insertion (Scheme 5C).^73^ In 2012, Molinaro and co-workers, also from Merck, developed a synthetic route towards MK-7246 (23, Scheme 5D), a CRTH2 antagonist, using [Ir(COD)Cl]_2_ catalyzed intramolecular N–H insertion to prepare intermediate 22 (R = H).^74^ The reaction was carried out at a 50 g scale. This strategy has also been used from researchers from AbbVie to synthesize azoles from aryl amidines and ketosulfoxonium ylides.^75^ In 2021, Ruck and co-workers from Merck disclosed the synthesis of the CRTH2 antagonist MK-1029 (24, Scheme 5D).^69^ The authors used the [Ir(COD)Cl]_2_ catalyzed intramolecular N–H insertion to prepare intermediate 22 (R = F) in a 64% yield from the corresponding sulfoxonium ylide. Lastly, very recently, Edgington-Mitchell and co-workers, in collaboration with Takeda Pharmaceutical, developed a series of activity-based probes containing a novel sulfoxonium ylide warhead (25, selective for cathepsin X) (Scheme 5E). The sulfoxonium ylides probes reacted *via* metal-free S–H insertion with cathepsins X cysteine residue, forming fluorescent adducts 26, which allowed measurement of its activity.^76,77^

**Scheme 6:**
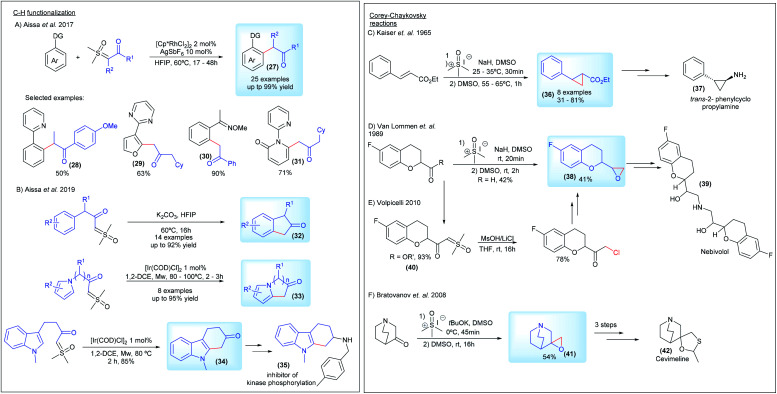
Sulfoxonium ylides in C–H functionalization and Corey–Chaykovsky reactions.

Regarding the C–H functionalization reactions, in 2017 Aïssa's group, in partnership with AstraZeneca, reported the cross-coupling reaction of sulfoxonium ylides with arenes and heteroarenes in the presence of a Rh(iii) catalyst (27, Scheme 6A). This transformation proceeds by directing group-mediated C(sp2)–H bond activation by pyridines, quinolines, pyrazoles, pyrimidines, oximes and *N*-methoxyamides to generate the desired products in yields of up to 99%. The more hindered bis-substituted sulfoxonium ylide derived from diethylsulfoxonium ethylide was also used as a substrate, affording the desired product 28 in a 50% yield.^26^ A few years later, the same group reported a metal-free and chemospecific strategy for intermolecular C–H functionalization of the sulfoxonium ylides (Scheme 6B). This transformation is enabled by the efficient combination of K_2_CO_3_ and HFIP (1,1,1,3,3,3-hexafluoro-2-propanol), leading to the important bicyclic ketones 32. However, this methodology fails with substrates containing the pyrrole nucleus and *N*-methyl indoles, but the authors found a direct alternative by using an [Ir(COD)Cl]_2_ catalyst to promote the intramolecular C–H functionalization reaction. In this case, the potential of the method was demonstrated by obtaining the key precursor for the synthesis of an inhibitor of kinase phosphorylation 35 from the C–H functionalization product of the indole substrate 34.^39^ Aïssa's group, working with AstraZeneca, also developed an important protocol enabling the synthesis of bis-substituted sulfoxonium ylides, using palladium chemistry. This work is presented in more detail in the section detailing the synthesis of the pro-chiral ylides.

Corey–Chaykovsky reactions have aroused interest in the industry as a crucial step to provide epoxide and cyclopropanes intermediates for the synthesis of pharmaceutical compounds (Scheme 6C–F).^78–82^ The first example, reported by Keiser and co-workers, from Smith Kline and French Laboratories, was a stereoselective approach towards the synthesis of *trans*-2-phenylcyclopropanecarboxylic acid derivatives 36 as potential intermediates to obtain the inhibitor of monoamine oxidase *trans*-2-phenylcyclopropylamine 37. The reaction of DMSM with esters of cinnamic acid and the related compounds afforded the substituted cyclopropanes in a 31–81% yield (Scheme 6C).^78^ In 1989, a patent assigned to Janssen Pharmaceutica N.V. described epoxidation reactions with sulfur ylides to synthesize 6-fluoro chroman epoxides (38, Scheme 6D). These compounds present the main structure of Nebivolol 39, an β-adrenergic blocker essential in the treatment of hypertension.^79^ Almost 20 years later, an alternative method using a halogenation strategy was presented by Volpicelli, from Zach Systems P.A., involving the synthesis of a 6-fluoro chroman-keto sulfoxonium ylide derivative (40, Scheme 6E).^80^ Another invention, patented in 2008 by Apotex Pharmachem Inc., described the reaction between 3-quiniclidinone and DMSM as a key step to generate the epoxide of 3-methylenequiniclidine (41, Scheme 6F). With three additional steps it can afford the precursor for cevimeline hydrochloride 42, used in the treatment of diseases of the central nervous system.^81^

**Scheme 7:**
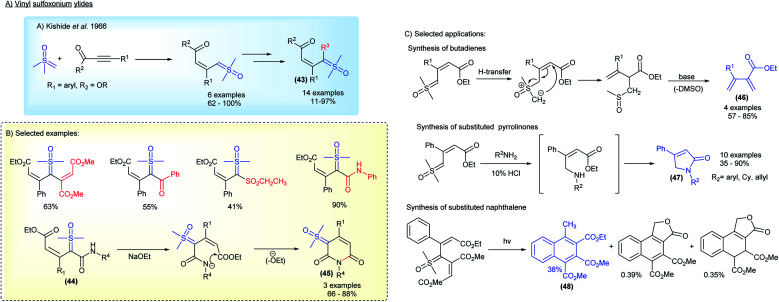
The synthesis and applications of vinyl sulfoxonium ylides.

Substituted sulfoxonium ylides distinct from the α-carbonyl series were also studied by research groups within the industry. For a decade, starting in 1966, the Central Research Laboratories in Japan investigated the synthesis and application of vinyl sulfoxonium ylides (Scheme 7).^83–90^ This class of sulfoxonium ylides, with adjacent α,β-unsaturated carbonyl groups, can be obtained from the reaction of DMSM with acetylenic compounds (Michael acceptors) (Scheme 7A). Although an example of vinyl sulfoxonium ylide was reported a year earlier by Trost,^78^ it was the Ide and Kishida's group that explored the generality of the method, successfully obtaining ylides derived from phenyl-propiolates, as well as performing subsequent functionalization, with acetylenes, isocyanates, benzoyl chlorides and sulfenes, to generate bis-substituted ylide structures 43 (Scheme 7B). The authors also reported the synthesis of 6-membered-N-heterocycles bearing a ylide group 45 by intramolecular cyclization of vinyl-carbamoyl sulfoxonium ylides 44 (3 examples, 66–88% yield).^86^ Among the main applications, the base-promoted preparation of butadienes 46, a cascade N–H insertion/intramolecular cyclization reaction to afford pyrrolinones 47, and the synthesis of naphthalenes 48*via* photochemical formation of carbenes should be highlighted (Scheme 7C).

## 3. Initial contributions to asymmetric reactions involving sulfoxonium ylides

**Scheme 8:**
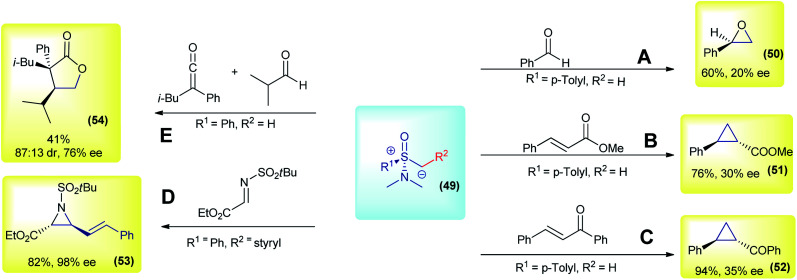
Representative examples of reactions with chiral aminosulfoxonium ylides.

The initial works on asymmetric reactions with sulfoxonium ylides relied on two different strategies: the use of chiral aminosulfoxonium ylides or enantioenriched substrates in combination with DMSM. In both cases, an enantioenriched reactant is used in equimolar amounts. The first example of a chiral sulfoxonium ylide dates back to 1968, in the pioneer work from Johnson's group. The authors employed a chiral aminosulfoxonium ylide (49, R_1_ = *p*-tolyl and R_2_ = H), prepared in four steps from the corresponding optically active sulfoxide, to perform asymmetric Corey–Chaykovsky reactions.^91^ In the following years, the same group published a series of works in which they expanded the methodology for other substrates, but in every case only low enantioselectivities were observed (max 20% enantiomeric excess (ee) for epoxidation and 43% ee for cyclopropanation).^91–94^ The main results (compounds 50, 51 and 52) are summarized in Scheme 8(A, B and C). Based on the same concept, in 2007, Adrien and co-workers prepared nine examples of alkenyl aziridine carboxylates by reacting allyl aminosulfoxonium ylides (prepared by deprotonation or fragmentation) with imino esters.^95^ The products were obtained in modest to high enantioselectivities for the *cis* isomer (47–98% ee). For example, product 53 was prepared in a 82% yield with 98% ee (Scheme 8D). Later, in 2014, Kerrigan used aminosulfoxonium ylides in an elegant strategy to prepare γ-lactones, from aldehydes and ketenes in a multicomponent reaction sequence.^96^ Under optimized conditions, 10 examples were prepared in yields of up to 65%, 95 : 5 dr and 79% ee. For example, compound 54 was prepared in a 41% yield, with 87 : 13 dr and 76% ee (Scheme 8E).

**Scheme 9:**
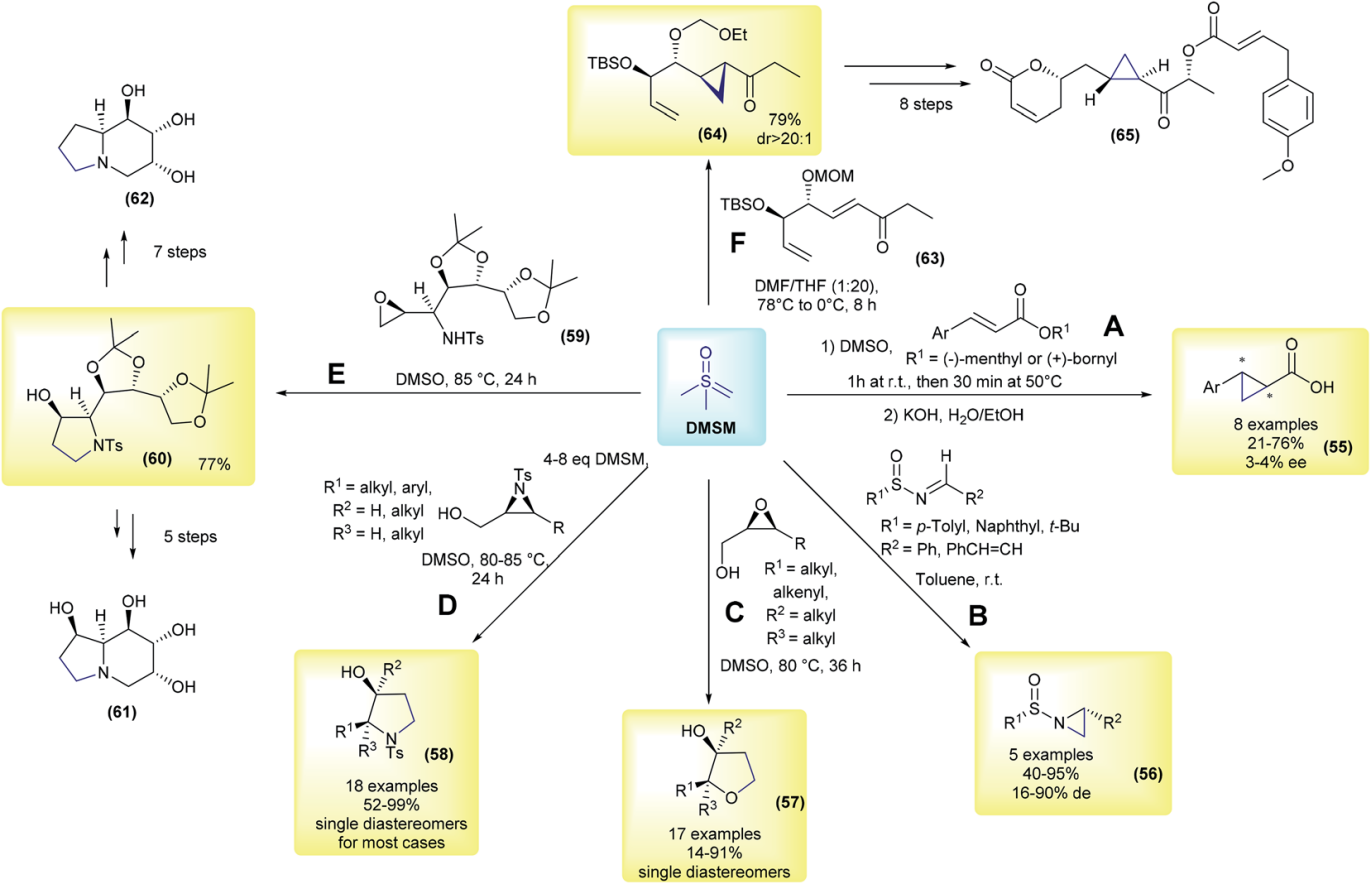
Representative examples of methodologies using DMSM and enantioenriched chiral substrates.

The first example using the chiral substrate approach was initially demonstrated by Kondo and co-workers, in 1966 (Scheme 9A).^97^ The authors used (−)-menthyl or (+)-bornyl β-arylacrylates as substrates for diastereoselective cyclopropanation in the presence of DMSM, followed by basic hydrolysis. Eight examples of arylcyclopropane carboxylic acids were prepared in yields ranging from 21–76%, but they had very low enantioselectivities (55, 3–4% ee). In the following years, several other reactions using DMSM in combination with different enantioenriched chiral substrates were published, these advances have been reviewed previously.^18,51,98^ This perspective will focus on a few representative and recent contributions. In 1996, Cruz and co-workers performed aziridination of chiral *N*-sulfinylimines.^99^ In their work, 5 aziridines (56) were prepared in good yields (40–95%) and with low to good diastereomeric excesses (16–90% de, Scheme 9B). In 2004, Borhan and co-workers developed a methodology to access 2,3-disubstituted tetrahydrofurans (57) from enantioenriched 2,3-epoxy alcohols and DMSM.^100^ 17 examples were synthesized with complete control of the stereochemistry, with *cis* epoxides yielding the corresponding *cis*-disubstituted tetrahydrofuran ring, while *trans*-epoxides furnished the *trans*-disubstituted rings (Scheme 9C). The authors found that using an excess of ylide and controlling the reaction concentration were crucial to the success of the reaction. In a following work, the same group applied this methodology to obtain diastereomerically and enantiomerically pure 2,3-disubstituted pyrrolidines from 2,3-aziridin-1-ols (58).^101^ These compounds are important heterocycles with a number of biological activities and are ubiquitously present in pharmaceuticals. In their work, 18 examples were reported with excellent yields and again with complete control of the stereochemistry (products were obtained as single diastereomers in most cases), as depicted in Scheme 9D. This strategy has also been applied in the total synthesis of bioactive compounds. In 2014, Tiwari and co-workers disclosed a divergent total synthesis of 1,6,8*a*-tri-epicastanospermine (61) and 1-deoxy-6,8*a*-di-epicastanospermine (62), from 59, involving an epoxide opening/intramolecular cyclization mediated by DMSM as one of the keys steps (Scheme 9E).^102^ In the same year, Hou and co-workers published the first total synthesis of (−)-brevipolide H (65), which is a bioactive natural product and a potential agent for treating human immunodeficiency virus (HIV).^103^ In their strategy, the authors conceived the cyclopropanation of intermediate 63 as one of the key steps. The cyclopropane 64 was obtained in a 79% yield and possessed a dr greater than 20 : 1 under optimized conditions. This intermediate was then converted to (−)-brevipolide H (65) after eight steps.

The contributions depicted in Schemes 8 and 9, as well as other studies,^18,51,98,104,105^ illustrate the importance and versatility of sulfoxonium ylides in asymmetric synthesis, but there are also challenges to be addressed. For example, all reactions described in this section followed the usual Corey–Chaykovsky reactivity, but the exploration of different reactivities is highly desirable. The preparation of more diverse and complex chiral ylides is also a topic that should be pursued. Comparatively, as described in the introduction, the diversity of the chiral sulfonium ylides is in great contrast to the limited variations of chiral sulfoxonium ylides previously reported. Finally, most reactions using the second approach (enantioenriched substrates) employ DMSM as the carbon source for 3-membered ring formation and homologations, therefore the use of more complex and/or substituted sulfoxonium ylides remains a challenge.^106^

## 4. Preparation of pro-chiral sulfoxonium ylides: importance and challenges

The term pro-chiral sulfoxonium ylide is dependent on the type of reaction being performed. A ylide can be pro-chiral for one reaction and not for another. In this perspective, this nomenclature will be used exclusively for reactions in which both an H and any other atom will be attached to their geminal position, resulting in a chiral molecule (for example, in X–H insertions; X = any atom ≠ H). Of course, if the reaction involves the addition of two different atoms at this geminal position, not including hydrogen, any mono-substituted ylide will be classified as pro-chiral (for example, in X–Y insertions; X and Y ≠ H).

**Scheme 10:**
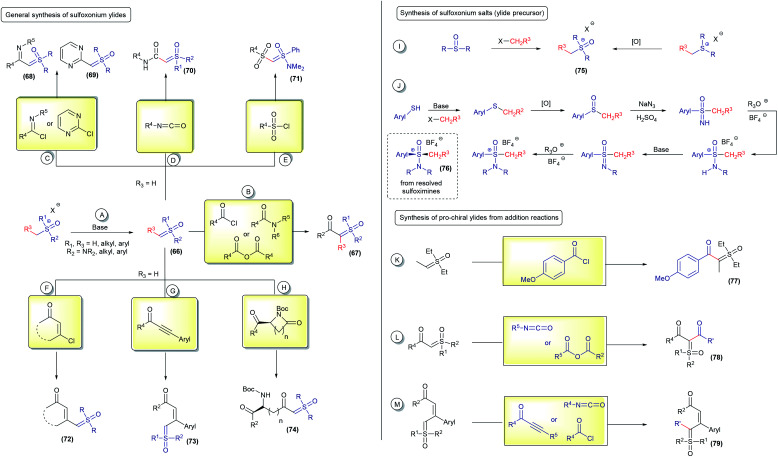
General protocols for the synthesis of sulfoxonium ylides.

The first syntheses of sulfoxonium ylides were accomplished by simple deprotonation of the corresponding sulfoxonium salts, using strong bases such as *t*BuOK or NaH (66, Scheme 10A). Unstabilized sulfoxonium ylides are normally obtained using this protocol (for example, dimethyl sulfoxonium methylide) and can be immediately used or stored as a solution at low temperature.^1^ From sulfoxonium methylides (R^3^ = H), several α-substituted sulfoxonium ylides can be obtained by nucleophilic additions to acid chlorides, anhydrides and amides (67, Scheme 10B),^107,108^ imidoyl chlorides and chloropyridmidines (68 and 69Scheme 10C),^109–111^ isocyanates (70, Scheme 10D),^17^ sulfonyl chlorides (71, Scheme 10E),^17^ β-chloro unsaturated ketones (72, Scheme 10F),^112–115^ propiolate derivates (73, Scheme 10G),^78,83^ and using the ring opening of lactams (74, Scheme 10H).^65^ These well-established procedures are efficient at providing a variety of sulfoxonium ylides, although there are still some important limitations to overcome. One of the biggest challenges consists of obtaining sulfoxonium salts with structural complexity. These salts, the main ylide precursors, are usually obtained by S-functionalization of sulfoxides (this works only for the methylation of dimethyl sulfoxide) or oxidation of the corresponding sulfonium salt (75, Scheme 10I), which are not general methods and have a limited scope. To date, only ylides containing R^3^ = H, Me, Ph and *n*Pr, different from those in the methylide series, have been efficiently obtained using route A from the deprotonation of trialkyl sulfoxonium salts.^116,117^ Very recently, Zeng^118^ described the preparation of a novel cyclic sulfoxonium ylide using a water-mediated intramolecular cyclization/oxidation of the sulfonium ylides, advancing the research on this topic. Nevertheless, there is still a lack of efficient methods to oxidize sulfonium ylides to acyclic sulfoxonium ylides. Regarding (dialkylamino)sulfoxonium ylides, precursor salts derived from sulfoximines can be prepared with enhanced structural diversification in a multistep synthetic route and also obtained as enantioenriched compounds by chiral resolution of the starting material (76, Scheme 10J).^17,94^ In both cases, sulfoxonium ylides with R^3^ ≠ H are less reactive for subsequent addition reactions to generate compounds of the type α,α′-bis-substituted (pro-chiral structures) (Scheme 10B). To the best of our knowledge, this step is limited to a few examples, R^3^ = Me by using diethyl sulfoxonium ethylide,^26^ carbonyl^63^ and propiolate^84^ (77–79, Scheme 10K–M).

**Scheme 11:**
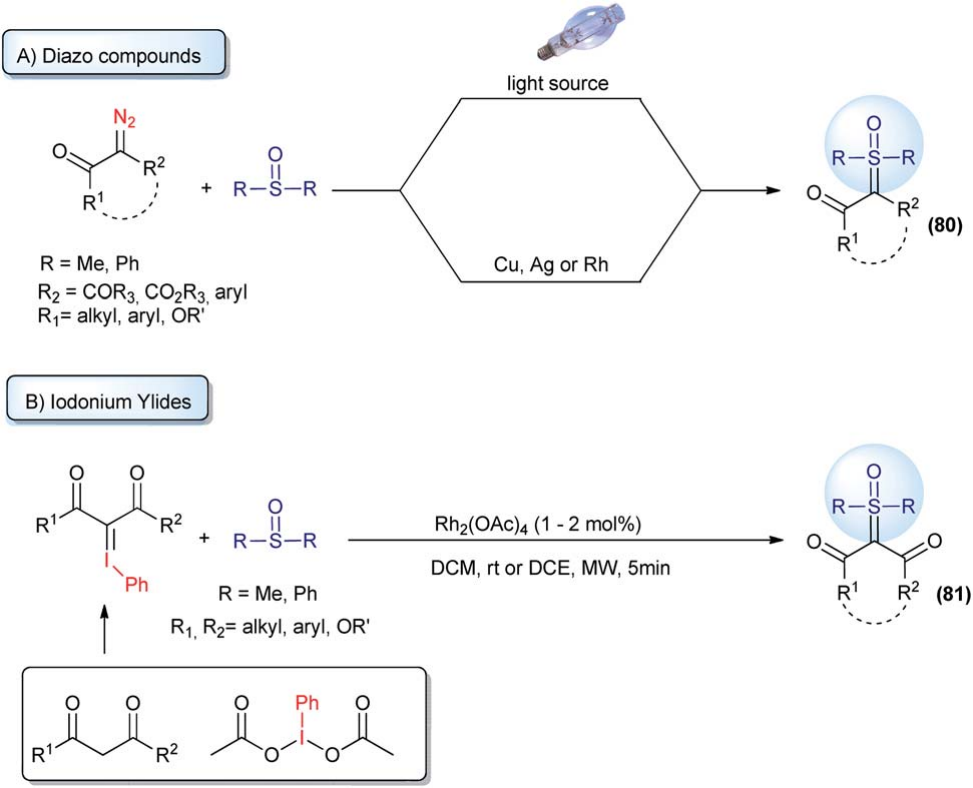
Synthesis of pro-chiral sulfoxonium ylides from the reaction of sulfoxides with diazo compounds or iodonium ylides.

Straightforward access to a wide scope of compounds, containing two different substituents (not including H) attached to the ylide carbon, is highly desirable. These structures are pro-chiral for X–H reactions (one of the most common transformations from these ylides) and allow access to enantioselective transformations that can go beyond the classic reactions for sulfur ylides, affording valuable products. In view of this, several works aim towards the synthesis of α,α′-bis-substituted sulfoxonium ylides have been reported since 1964 (ref. 64) and it remains an active research topic. The first general protocol was developed using diazo compounds as reagents.^119,120^ This transformation is based on the decomposition of the diazo substrates to generate carbenes in the presence of sulfoxides. The carbene intermediates can be accessed using two pathways: (i) exposure of the starting material to UV radiation from a high-pressure mercury lamp; or (ii) to the presence of copper or silver salts (Scheme 11A). Recently, some improvements have been made regarding this synthetic strategy. The use of visible light (blue LED) instead of UV radiation proved to be very efficient.^121,122^ In addition, catalytic amounts of copper powder or rhodium complexes were employed, reducing the amount of metal catalyst to promote the reaction.^38,123^ Diazo substrates containing donor–acceptor and acceptor–acceptor substituents, with acceptor = carbonyl and donor = aryl, are well tolerated by this strategy and the products are provided in high yields. It is worth noting that there are some concerns about the large scale applications of diazo compounds in organic reactions, mainly owing to the safety issues resulting from rapid exothermic reactions and gas release. In addition, the use of diazo substrates containing an alkyl substituent, instead of aryl, have not been reported. These drawbacks led the scientific community to investigate synthetic alternatives.

Iodonium ylides are substrates that are also capable of reacting with sulfoxides in the presence of metal catalysts to afford sulfoxonium ylides (81, Scheme 11B).^124,125^ Despite being an alternative to the use of diazo compounds, the scope of this transformation is restricted to the production of dicarbonyl sulfoxonium ylides. Dicarbonyl iodonium ylides overcome the stability and solubility issues normally associated with iodonium ylides. In this case, the substrate can be isolated prior to use or generated *in situ* from phenyliodonium diacetate (PhI(OAc)_2_) and active methylene compounds.

**Scheme 12:**
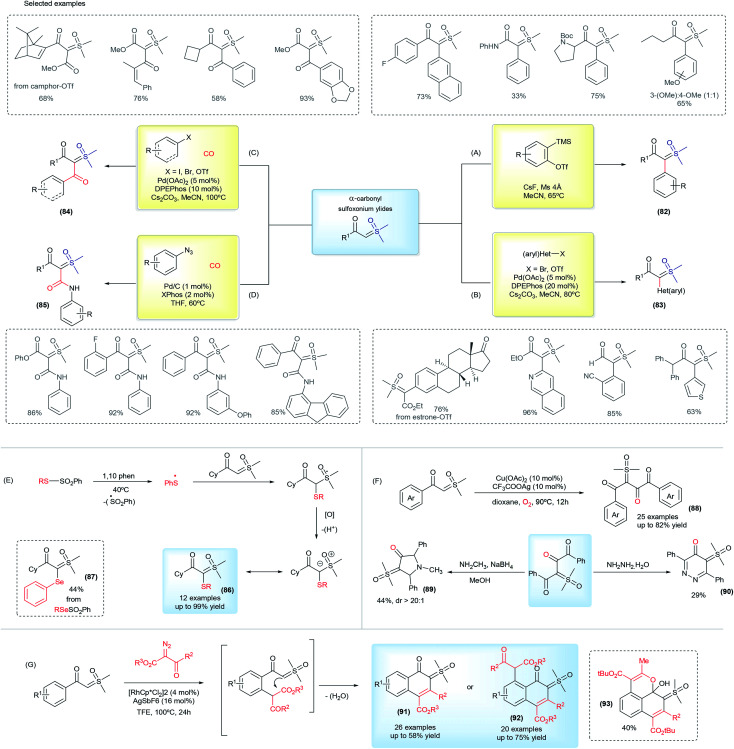
The synthesis of bis-substituted sulfoxonium ylides and selected examples.

In 2017, Burtoloso and co-workers took the initial step towards an effective pathway to obtain pro-chiral sulfoxonium ylides (82) from readily available substrates and without the use of any diazo reagent (Scheme 12A).^126^ The methodology consists of the arylation reaction of α-carbonyl sulfoxonium ylides using aryne chemistry, providing 40 examples of the product 82, containing the substituents α-aryl-α-carbonyl in good yields (up to 85%). Sulfoxonium ylides obtained using this methodology are structurally-dependent on the availability of the aryne precursors and the reaction can show regioselectivity issues with the use of unsymmetrical substrates. This method also failed when pyridinium and non-aromatic aryne precursors were applied. Afterwards, Aïssa and co-workers overcame some of these limitations by developing versatile palladium-catalyzed cross coupling reactions of aryl bromides or triflates with α-carbonyl sulfoxonium ylides, providing α-(hetero)aryl functionalized products (83, Scheme 12B).^127,128^ This reaction works with a broad range of structurally diverse aryl and heteroaryl substrates, which are applied in the synthesis and post functionalization of drug candidates and natural compound analogues. Palladium cross coupling reactions are also efficient for the production of dicarbonyl sulfoxonium ylides (84) by carbonylation of aryl halides or azides with sulfoxonium ylides, conducted under CO_(g)_.^129,130^ This transformation is efficient with aryl and heteroaryl substrates, and also with vinyl halides and triflates, affording the sulfoxonium bis-substituted ylides with a keto and ester group (Scheme 12C). When azides are applied as coupling partners, it can also provide α-carbonyl-α′-amide sulfoxonium ylides (85, Scheme 12D).

Recently, several methodologies have been developed to prepare bis-substituted sulfoxonium ylides containing substituents that are different from the common aryl and carbonyl groups. The synthesis of α-keto, α′-thioaryl sulfoxonium ylides (86) can be accomplished by exposing cyclohexyl-keto sulfoxonium ylides to the presence of thiosulfonates and 1,10-phenanthroline, *via* a radical insertion reaction.^131^ This reaction can also provide the selenium-substituted sulfoxonium ylide (87) from phenylbenzenesulfonoselenoate in a 44% yield (Scheme 12E). Homocoupling of the keto sulfoxonium ylides in the presence of a copper catalyst in aerobic conditions provides the α,α,β-tricarbonyl sulfoxonium ylides (88).^132^ The ylide carbon on these structures is one of the substituents and is composed of two vicinal carbonyls groups. In addition, these products can undergo subsequent transformations with methylamine or hydrazine to provide previously unreported N-heterocyclic sulfoxonium ylides in moderate yields (89 and 90, Scheme 12F). Another example of cyclic sulfoxonium ylide synthesis is depicted in Scheme 12G.^133^ These ylides were obtained after a Rh(iii)-catalyzed C–H activation/intramolecular cyclization cascade reaction from keto sulfoxonium ylides and diazo carbonyl compounds. This transformation produces a broad scope of highly functionalized naphthalenones, bearing a sulfoxonium ylide moiety, in 14–75% yields (91 and 92). An example (93) with a benzo[de]chromene nuclei was also demonstrated when *tert*-butyl 2-diazo-3-oxobutanoate was used as a substrate.

Among all the methodologies presented, there are still countless structural variations of sulfoxonium ylides that have not been accomplished as yet. Furthermore, there is a lack of prochiral ylides containing alkyl substitutes. These wide ranging possibilities, together with the rapid advance in asymmetric reactions with sulfoxonium ylides described in recent years, make the development of methodologies for obtaining pro-chiral sulfoxonium ylides an ambitious field in organic synthesis.

## 5. Recent achievements relating to efficient enantioselective transformations involving sulfoxonium ylides

**Scheme 13:**
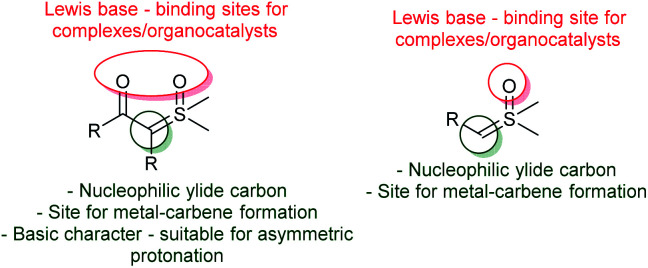
Possible modes for enantioselective catalysis involving sulfoxonium ylides.

This topic will focus on the recent catalytic enantioselective methods reported using sulfoxonium ylides. In contrast to sulfonium ylides, in which several methodologies for catalytic and enantioselective transformations were developed in the 1990s and early 2000s, the number of reported catalytic asymmetric reactions using sulfoxonium ylides is still scarce.^18,51,98^ Considering the sulfoxonium ylide general structure, there are a few possible modes of enantioselective catalysis that can be highlighted (Scheme 13). First, oxygens from the carbonyl groups and sulfoxide moiety of the ylide can act as Lewis bases, furnishing binding sites for chiral metal complexes or organocatalysts, such as chiral hydrogen-bond donors. Sulfoxonium ylides can also form metal-carbenes owing to the nucleophilic carbon, allowing the possibility for enantioselective transformations if chiral complexes are used. Finally, the nucleophilic carbon can also be protonated by Brønsted acids, making asymmetric protonation possible if chiral Brønsted acids are employed.

**Scheme 14:**
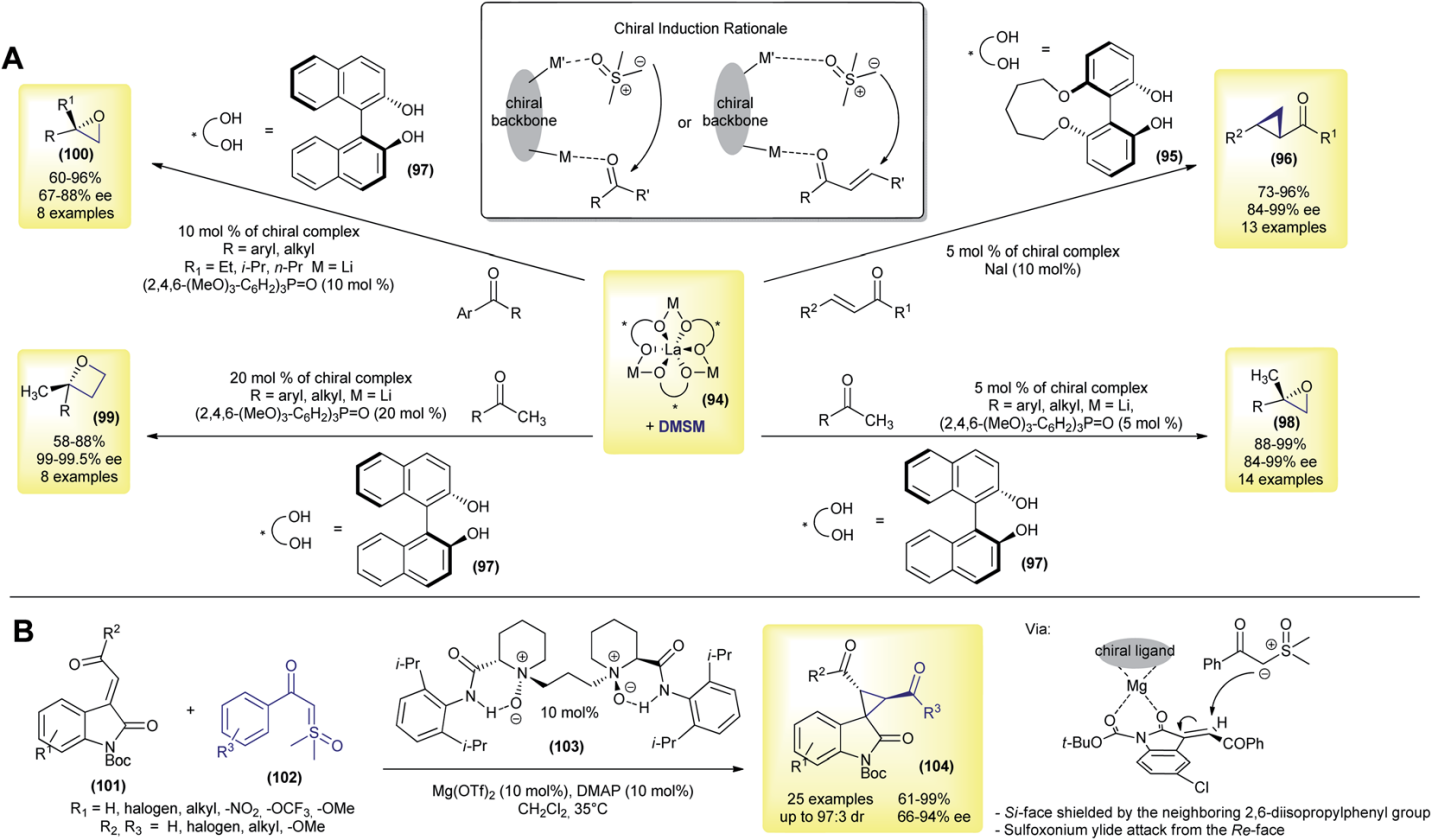
Asymmetric catalytic Corey–Chaykovsky methodologies using sulfoxonium ylides.

The first methodology using sulfoxonium ylides in catalytic asymmetric transformations was published in 2007, by Shibasaki's group.^134^ In their work, the authors performed a catalytic asymmetric Corey–Chaykovsky cyclopropanation of enones with DMSM promoted by a La–Li_3_-(biphenyldiolate)_3_ + NaI system. Under optimized conditions, 13 *trans*-cyclopropanes (96) were prepared in good yields (73–97%) and demonstrated an excellent enantioselectivity (84–99% ee, Scheme 14A). The use of NaI as an additive, as well as biphenyldiol 95, played a key role in achieving high enantioselectivity. In the following year the same group expanded their methodology by using a similar catalytic system (94 + 97) and performing enantioselective Corey–Chaykovsky epoxidations of aryl and alkyl methylketones.^135^ 13 2,2′-disubstituted epoxides (98) were prepared in excellent yields and with a good enantioselectivity in the presence of a triarylphosphine oxide as an additive (88–99% yield and 91–97% ee, Scheme 14A). In 2009, Shibasaki's group presented a one-pot asymmetric methodology to transform ketones into 2,2′-disubstituted oxetanes (99).^136^ The authors prepared eight examples in good yields (58–88%) and with excellent enantioselectivities (99–99.5 ee) (Scheme 14A).

In the last contribution of this series, the group further expanded the methodology with eight examples of 2,2′-disubstituted epoxides (100) from alkyl (ethyl, isopropyl and *n*-propyl) aryl ketones, in good yields (60–96%) and with good enantioselectivities (67–88% ee).^137^ These reactions are believed to occur *via* dual control from a heterobimetallic chiral complex, with a simultaneous interaction with the ketone and DMSM, as highlighted in Scheme 14.

In 2018, Feng and co-workers disclosed a novel enantioselective cyclopropanation of 3-alkenyl-oxindoles with sulfoxonium ylides.^106^ In contrast to all of the previously reported papers, the authors were able to use keto sulfoxonium ylides over simple DMSM (Scheme 14B).^106^ Using a combination of a chiral *N*,*N*′-dioxide ligand (103) and Mg(OTf)_2_ as a catalyst, 25 spiro-cyclopropyl oxindoles (104) were obtained in good yields (61–99%), with an excellent dr (up to 97 : 3 dr) and good to excellent enantiomeric excesses (66–94% ee). Interestingly, when the authors used DMSM as a ylide source under the reaction conditions, the cyclopropane product was formed in only 35% yield and 57 : 43 dr (16% ee and 5% ee, respectively). The Boc protecting group is the key to achieving a high enantioselectivity. In this case, the authors hypothesized that the attack of sulfoxonium ylide on the metal-chiral ligand–substrate complex would occur from the least hindered face.

**Scheme 15:**
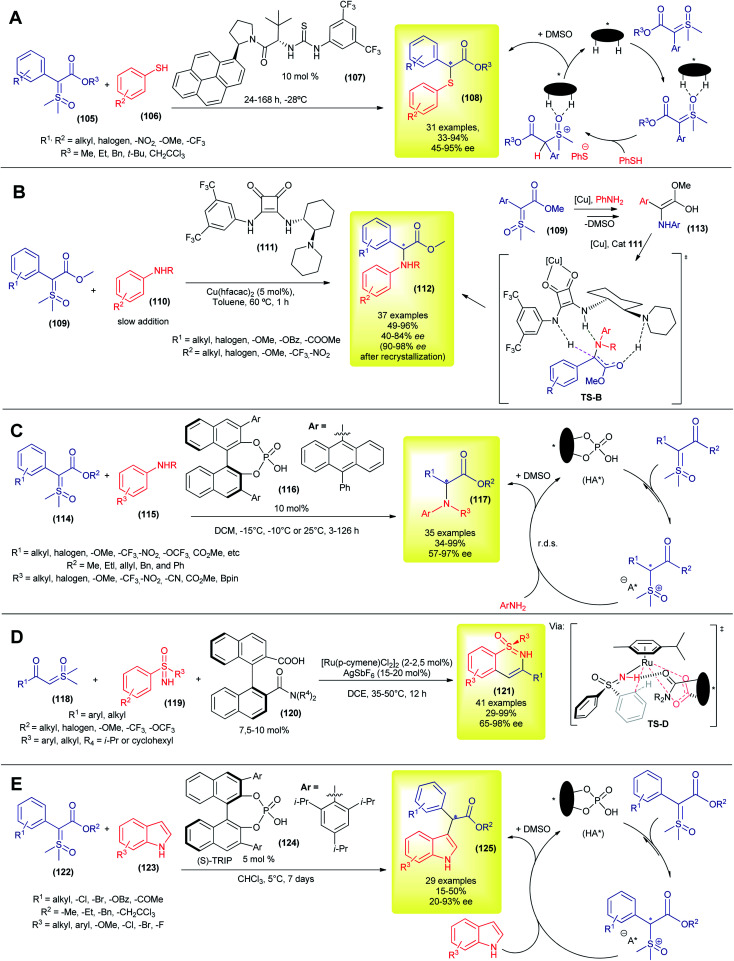
Asymmetric catalytic X–H insertion with sulfoxonium ylides.

Despite these great contributions, the aforementioned methodologies are still based on the Corey–Chaykovsky reactivity. The first example of asymmetric transformation with sulfoxonium ylide that was not based on Corey–Chaykovsky reactivity was only disclosed in 2020, by Burtoloso's group. In their work, they took advantage of the activity of the aryl thiols and reported the first protocol for asymmetric S–H insertion into pro-chiral sulfoxonium ylides (Scheme 15A).^38,138^ The authors screened several types of dual hydrogen bond donor catalysts, with Jacobsen thiourea (107) furnishing the best enantioselectivity. Using this methodology, 31 α-carbonyl thioethers (54) were synthesized, in moderate to excellent yields (33–94%) and enantioselectivities (45–95% ee, Scheme 15A). The temperature control (optimum temperature is −28 °C) was decisive for good enantioselection, and so was the solvent. The authors combined nuclear magnetic resonance (NMR) studies and density functional theory (DFT) calculations to propose a mechanism sequence in which the organocatalyst initially interacts with the oxygen of the S=O bond and this complex is then protonated by the thiol in the enantiodetermining step (Scheme 15A). This chiral ion pair then collapses, furnishing the thiolate substitution product (108) and DMSO.

Although the metal-carbene mediated X–H insertion reaction from sulfoxonium ylides has been known since 1993, it was only in 2021 that the first highly enantioselective carbene mediated transformation using sulfoxonium ylide was published, by Burtoloso's group (Scheme 15B).^139^ Using a copper-bifunctional-squaramide cooperative catalytic system, the authors synthesized 37 α-arylglycine esters (112) in 49–96% yields and 40–84% ee values (90–98% ee after one recrystallization). In contrast to the previously reported novel enantioselective insertion methodology, which requires long reaction periods and prolonged cooling, this methodology works best at 60 °C, and the reaction reaches completion within 1 h. In addition, it utilizes the inexpensive copper metal and does not require special conditions (benchtop procedure). To account for the enantioselectivity, the authors hypothesized a mechanism (Scheme 15B) similar to the one studied in detail by Zhou and co-workers in the asymmetric N–H insertion reaction between alkylamines and diazocompounds.^140^ After the formation of electrophilic copper-carbene and the subsequent attack of aniline, the copper-organocatalyst complex coordinated with the enol intermediate 113, forming a transition state TS-B. Asymmetric protonation of this chiral TS-B gives rise to the enantioenriched product 112. This strategy allows straightforward access to a variety of bioactive compounds.^139^ In 2020, Sun and co-workers developed a protocol for asymmetric N–H insertion, using a combination of prochiral α-carbonyl sulfonium ylide and arylamines.^57^ As discussed previously, sulfonium ylides present some disadvantages when compared to the sulfoxonium ylides, but nonetheless were suitable substrates for this chiral phosphoric acid promoted reaction. The authors prepared 35 α-amino carbonyl compounds (mainly methyl α-amino ketones) with yields and enantioselectivities in the 40–94% and 78–99% ranges, making this and Burtoloso's methodologies complementary to each other.

Later in 2021, the same group, led by Sun, developed their own methodology to access α-arylglycine esters (117), using chiral phosphoric acids as organocatalysts in a metal-free approach to asymmetric N–H insertion with sulfoxonium ylides (Scheme 15C).^141^ Under optimized conditions, they were able to prepare 35 examples, mostly in good yields (34–99%) and with moderate to excellent enantioselectivities (57–97% ee). In a similar way to their catalytic system using sulfonium ylide,^57^ the protonation of sulfoxonium ylide is reversible, followed by a rate determining nucleophilic attack by aniline. The chiral phosphate anion drives the stereochemistry outcome in the C–N bond formation step, *via* dynamic kinetic resolution.

Also in 2021, Shi and co-workers reported the first enantioselective C–H insertion/annulation with sulfoxonium ylides (Scheme 15D).^142^ Using sulfoximines as substrates and sulfoxonium ylides as coupling partners in a chiral ruthenium-complex catalyzed reaction, the authors synthesized 41 enantioenriched cyclic sulfoximines (121) *via* desymmetrization, kinetic resolution and parallel kinetic resolution strategies. In most cases the enantioselectivity was excellent (up to 98% ee). The authors performed a few experiments to gain insights into the reaction mechanism and the high resolution mass spectrometry (HRMS) evidence points to the formation of a five-membered ruthenacycle transition state (TS-D).

Very recently, the first example of an organocatalytic enantioselective formal C–H insertion between sulfoxonium ylide and indoles (C3 position) was disclosed (Scheme 15E), by Burtoloso's and Mattson's groups, working in collaboration.^143^ Using chiral phosphoric acid ((*S*)-TRIP, 124) catalysis, the researchers were able to obtain 29 formal C–H insertion products (125) with enantioselectivities in the range of 20–93% ee and low to moderate yields (15–50%). The reactions proceeded without the need for nitrogen protection in the indole. The observed enantioselectivity reached the optimum value at 5 °C, but long reaction periods were required (7 d). The solvent choice and ester moiety in the ylide dramatically influenced the enantioselectivity. It is worth mentioning that the racemic version of this reaction is also unprecedented with sulfoxonium ylides and furnished better reaction yields at room temperature. The proposed catalytic cycle was very similar to the one displayed in Scheme 15C.

After displaying the published methodologies for the asymmetric catalytic transformation of the sulfoxonium ylides it becomes clear that more intense exploration of this area has only occurred very recently. Important frameworks were prepared with highly efficient and straightforward strategies. However, there are still many challenges as these methodologies are still limited to specific substrates and conditions. As discussed previously, there are also limitations in the preparation of pro-chiral ylides, but the rapidly growing interest in sulfoxonium ylides should remedy this current limitation. In summary, the unique reactivity of the sulfoxonium ylides, combined with their stability, nucleophilic character and propensity to form metal carbenes under appropriate conditions makes this class of compounds ideal platforms for the development of asymmetric and more complex transformations.

## 6. Conclusions and perspectives

This perspective discussed asymmetric transformations involving sulfoxonium ylides in detail, culminating in very recent developments relating to catalytic enantioselective variants. A brief introduction was accompanied by deep discussion of the importance and utilization of sulfoxonium ylides in industry. Although sulfur ylides are not substitutes for diazo compounds in all reactions involving the latter, there are many transformations that can be accomplished efficiently using both classes of compounds. For these transformations, the choice has been the use of the safer sulfur ylides (especially for large-scale preparation and in industry). An interesting point that was highlighted in several parts of this perspective is the comparison between sulfonium and sulfoxonium ylides. Although it can be contradictory (sulfoxonium ylides are claimed to be more interesting substrates than the sulfonium examples owing to many factors, but the chemistry of the latter has advanced more quickly), there is a reason for this difference in advances: structurally diverse sulfoxonium substrates are more difficult to obtain when compared to sulfonium ylides. Once novel methods appear and this limitation is circumvented, the chemistry will be boosted. The absence of a vast array of sulfoxonium substrates, including more complex pro-chiral sulfoxonium ylides, added to the reduced reactivity, also influenced the slow discovery of asymmetric versions. Although enantioselective S–H and N–H insertion reactions are now efficient, C–H ones, for example, would still benefit from development. Enantioselective halogenation and dihalogenation reactions involving sulfoxonium ylides, as well as the use of these ylides in bioconjugate chemistry, are some of the innovative topics in this area of chemistry. With respect to the synthesis of novel sulfoxonium ylides, methods that will efficiently alkylate α-carbonyl sulfoxonium ylides (arylation methods are already a reality) and allow the preparation of different sulfoxonium salts compared to trimethyl sulfoxonium examples are highly desirable.

## Author contributions

A. C. B. B. conceived the topic and structure of the perspective. C. D. A. C. and L. G. F. equally collected the references, wrote the main text, and prepared the schemes and figures. A. C. B. B. reviewed the perspective and added his personal view about the theme at some points in the text.

## Conflicts of interest

There are no conflicts to declare.

## Supplementary Material
